# Associations of the TNF-alpha -308 G/A, IL6 -174 G/C and AdipoQ 45 T/G polymorphisms with inflammatory and metabolic responses to lifestyle intervention in Brazilians at high cardiometabolic risk

**DOI:** 10.1186/1758-5996-4-49

**Published:** 2012-11-24

**Authors:** Maira LR Curti, Milena M Pires, Camila R Barros, Antonela Siqueira-Catania, Marcelo Macedo Rogero, Sandra RG Ferreira

**Affiliations:** 1 Department of Nutrition, School of Public Health, University of Sao Paulo, Av. Dr. Arnaldo, São Paulo, SP, 715, Brasil

**Keywords:** Polymorphisms, Intervention, Lifestyle, Cardiometabolic risk

## Abstract

**Background:**

Cytokines secreted by the adipose tissue influence inflammation and insulin sensitivity, and lead to metabolic disturbances. How certain single-nucleotide polymorphisms (SNPs) interfere on lifestyle interventions is unclear. We assessed associations of selected SNPs with changes induced by a lifestyle intervention.

**Methods:**

This 9-month intervention on diet and physical activity included 180 Brazilians at high cardiometabolic risk, genotyped for the TNF-α -308 G/A, IL-6 -174 G/C and AdipoQ 45 T/G SNPs. Changes in metabolic and inflammatory variables were analyzed according to these SNPs. Individuals with at least one variant allele were grouped and compared with those with the reference genotype.

**Results:**

In the entire sample (66.7% women; mean age 56.5 ± 11.6 years), intervention resulted in lower energy intake, higher physical activity, and improvement in anthropometry, plasma glucose, HOMA-IR, lipid profile and inflammatory markers, except for IL-6 concentrations. After intervention, only variant allele carriers of the TNF-α -308 G/A decreased plasma glucose, after adjusting for age and gender (OR 2.96, p = 0.025). Regarding the IL6 -174 G/C SNP, carriers of the variant allele had a better response of lipid profile and adiponectin concentration, but only the reference genotype group decreased plasma glucose. In contrast to individuals with the reference genotype, carriers of variant allele of AdipoQ 45 T/G SNP did not change plasma glucose, apolipoprotein B, HDL-c and adiponectin concentrations in response to intervention.

**Conclusion:**

The TNFα -308 G/A SNP may predispose a better response of glucose metabolism to lifestyle intervention. The IL-6 -174 G/C SNP may confer a beneficial effect on lipid but not on glucose metabolism. Our findings reinforce unfavorable effects of the AdipoQ 45 T/G SNP in lipid profile and glucose metabolism after intervention in Brazilians at cardiometabolic risk. Further studies are needed to direct lifestyle intervention to subsets of individuals at cardiometabolic risk.

## Introduction

Obesity-related diseases are major public health problems. Intervention programs have been based on dietary changes and physical activity aiming at controlling weight gain and prevent metabolic disturbances. Adipose tissue is an important source of cytokines capable to trigger inflammatory mechanisms and generate insulin resistance, involved in the genesis of these diseases [1].

Apart from the role of environmental determinants, also genetic factors predispose to obesity and may influence the response to interventions on lifestyle. Heterogeneous responses to lifestyle interventions should be at least in part controlled by genetic factors [2]. Single-nucleotide polymorphisms (SNPs) were described in association with the risk for obesity and metabolic diseases [3], but how certain SNPs could interfere on the process of weight loss is less investigated.

The tumor necrosis factor-α (TNF-α) SNP at position -308 G/A [4] has been associated with obesity risk [5] and increased C-reactive protein (CRP) concentrations [6]. A positive correlation between the adipose tissue mass and expression of the gene that encodes TNF-α was described [7]. The role of this adipocytokine in inflammatory response is well-established [8]. Also, TNF-α deteriorates insulin signaling and reduces glucose uptake [9], mediating the relationship of insulin resistance with manifestations of the metabolic syndrome (MS) [10]. A functional impact of G and A alleles in the synthesis of TNF-α is controversial [11,12].

Interleukin-6 (IL-6) has also been associated with cardiometabolic diseases; its concentrations were shown to be predictive of type 2 diabetes (T2D) [13]. Despite being essential for reducing acute inflammatory processes, in chronic inflammatory states may assume a pro-inflammatory role [14]. Adipose tissue is one of the most important sources of IL-6 secretion, and visceral tissue secrets 2 to 3 times more IL-6 than the subcutaneous tissue [13]. Associations of the −174 G/C polymorphism with increased adiposity, inflammation and metabolic disturbances have been investigated [15]. Carriers of C allele seem to have different cardiometabolic effects depending on body mass index (BMI). The −174 G/C was associated with T2D in overweight individuals but not in eutrophic ones [16]. In contrast, joint analysis, based on data from 17 studies, suggested an association of IL-6 G/C genotype with fasting glucose levels independently of the BMI [17]. Moreover, C allele carriers have shown greater IL-6 concentrations, leading to an increase in circulating CRP [18-20]. On the other hand, the reference genotype (G allele) was associated with higher triglycerides, very low density lipoprotein, plasma glucose and lower high density lipoprotein (HDL-c) concentrations than C allele carriers [21].

Adiponectin plays a role in energy homeostasis regulation, lipid and glucose metabolism and insulin sensitivity [22,23]. This adipocytokine is also known by its anti-atherogenic and anti-inflammatory properties. Low concentration of adiponectin was found in T2D [24] and coronary heart disease [25]. The AdipoQ 45 T/G polymorphism was shown to be associated with hypoadiponectinemia and risk of T2D in different populations [26-28]. The STOP-NIDDM found a 1.8 times greater risk for T2D in carriers of the variant allele [29]. Also, associations of this SNP with other diseases included in the spectrum of the MS have been reported [30-32].

To date, relatively few studies have investigated the associations of common variants in the genes coding for TNF-α, IL-6 and adiponectin with their plasma concentration changes following interventions on lifestyle. Assuming that part of the heterogeneous response to lifestyle interventions among populations may be explained by genotypic factors, we investigated whether certain inflammation related-polymorphisms influence the response profile of Brazilian individuals. This study assessed the association of the TNF-α -308 G/A, IL-6 -174 G/C and AdipoQ 45 T/G polymorphisms with changes in anthropometric, metabolic and inflammatory biomarkers following a lifestyle intervention in Brazilians at high cardiometabolic risk.

## Methods

This was a 9-month intervention study on lifestyle for prevention of T2D carried out during 2009 and 2010. The study protocol was approved by the local ethical committee and individuals provided informed written consent before entering the study. Individuals aged from 18 to 80 years were screened at Healthcare Unit of the School of Public Health, University of São Paulo, Brazil. Those with at least one risk factor were invited to a clinical examination and laboratory tests including a 75-gram oral glucose tolerance test. Inclusion criteria were the presence of prediabetes (impaired fasting glucose or impaired glucose tolerance according to the American Diabetes Association [33] or MS according to International Diabetes Federation criteria for Latin-American populations [34]. For the purpose of this study, two categories of skin colors was defined – white and non-white – being the latter composed of mulattos and blacks. Asian descendants were excluded. Those with a medical history of neurological or psychiatric disturbances, thyroid, liver, renal and infectious diseases were excluded.

The intervention on lifestyle consisted of medical consultations every three months, where counseling for changing living habits was given. Our intervention goals were similar to those previously proposed [1,2]. Individuals also attended group sessions, where topics on healthy diet, physical activity and psychosocial stress management were discussed with a multi-professional team. At the end of the ninth month, examinations were repeated. Compliance rate to the intervention program did not differ between subgroups of individuals stratified according to the genotypes.

A total of 301 individuals were eligible and 180 agreed to participate. Thirty-seven participants lost to follow-up and eight DNA samples with low quality that resulted in poor genotyping calls were excluded.

Dietary data were collected using 24-hour food recalls by trained nutritionists. Physical activity level was assessed by the long version of the international Physical Activity Questionnaire [35]. Individuals that achieved 150 min per week of total physical activity were considered active.

Height was measured using a fixed stadiometer, weight was obtained from individuals wearing light clothes and no shoes on a Filizola digital scale and BMI was calculated. Abdominal circumference was measured at the midpoint between the bottom of the rib cage and above the top of the iliac crest during minimal respiration.

Venous blood samples were collected after overnight fasting. Plasma glucose was measured by the glucose-oxidase method and lipoproteins determined enzymatically by automatic analyser. Aliquots were frozen at −80°C for further determinations of apolipoproteins (Apo), inflammatory markers and for genotyping. Apo A1 and B concentrations were determined using turbidimetry. CRP, IL-6 and TNF-α concentrations were determined by immunoenzyme chemiluminescent assay (Immulite, Diagnostic Products Corporation, Los Angeles, CA, USA). Serum insulin was determined by immunometric assay using a quantitative chemiluminescent kit (AutoDelfia, Perkin Elmer Life Sciences Inc, Norton, OH, USA). Adiponectin was measured by ELISA (Linco Research, St. Charles, Missouri, USA). Whole blood was used for DNA extraction for determination of the SNPs.

### Genotyping

Genomic DNA was extracted from whole blood using the *salting out* method [36]. After extraction, the viability of the extracted material was visualized in 1% agarose gel and concentration was obtained by spectrophotometer Nanodrop 8000 (Thermo Scientific, Waltham, MA, USA). Genotyping of the TNF-α -308 G/A (rs1800629), IL-6 -174 G/C (rs1800795) and AdipoQ 45 T/G (rs 2241766) polymorphisms was performed using an allele specific polymerase chain reaction (PCR) or amplification refractory mutation [37]. Detection of the products was made using fluorescence resonance energy [38]. PCRs were carried out with ArrayTape instrumentation, and allele calls were generated based on the clustering of fluorescent signals. Only genotypes with a level of confidence ≥90% were included in the analysis. Moreover, a total of 30 duplicate samples were included in an attempt to check for genotyping errors. The genotype success rates for TNF-α -308 G/A, IL-6 -174 G/C and AdipoQ 45 T/G were 98%, 99% and 99%, respectively.

### Statistical analysis

Data were expressed by means and standard deviations (SD) or medians and interquartile intervals. The normality of the distribution of each variable was analyzed by Kolmogorov-Smirnov test. Log-transformation was performed to obtain normal distribution of skewed variables (insulin, adiponectin, IL-6 and CRP). Student *t* test (or Wilcoxon) was used to compare means of variables between groups with and without polymorphisms. Chi-square test was used to evaluate the Hardy-Weinberg equilibrium (p > 0.05). Stepwise logistic regression analysis was performed to evaluate the impact of genotypes on clinical variables changes, dichotomized by using arbitrary criteria (i.e. ≥1 SD change) for improvement in variables from baseline to 9-month examination. Each clinical improvement (dependent variable) was used in a separate model. Presence of the variant allele was considered the independent variable of main interest, adjusted for sex and age. Variables showing a p-value <0.20 in crude analysis entered the multiple models.

Statistical analyses were performed using the SPSS 17.0 (SPSS Inc., Chicago, IL, USA) for Windows. Statistical significance was assumed when *p* values were < 0.05.

## Results

Among 138 individuals, 66.7% were women and 30.4% non-white; the mean age of the sample was 56.5 (± 11.6) years and BMI was 30.3 (± 5.6) kg/m^2^. A total of 62.3% had prediabetes and 90.6% MS. After intervention, mean values of energy intake reduced and total physical activity elevated significantly considering the entire sample (Table 1), as well as the subsets of individuals stratified by the presence of the polymorphisms (data not shown). The proportion of active individuals at the ninth month was higher than at baseline (52.2 *versus* 55.6%, p <0.05). Significant improvement in anthropometric variables, plasma glucose, lipid profile and inflammatory markers was observed, except for IL-6 concentrations.

**Table 1:** Mean (SD) or median (interquartile interval) values of variables at baseline and after 9 months of lifestyle intervention in total sample

	**Baseline**	**9 months**	***p***
Energy intake (kcal/day)	1814 (673)	1530 (558)	0.008
Total physical activity (min/wk)	186 (60, 262)	221 (70, 303)	0.007
Weight (kg)	78.6 (15.5)	76.7 (15.4)	0.005
Waist circumference (cm)	99.2 (12.3)	98.1 (12.0)	0.005
Fasting plasma glucose (mg/dL)	99.5 (11.5)	96.7 (12.5)	0.018
2-h plasma glucose (mg/dL)	119.3 (28.3)	113.1 (29.3)	0.009
Triglycerides (mg/dL)	155.0 (65.5)	151.3 (71.5)	0.491
Total cholesterol (mg/dL)	202.0 (43.6)	198.0 [3,43]	0.238
HDL-c (mg/dL)	42.5 (12.4)	46.7 (12.5)	0.001
LDL-c (mg/dL)	128.0 (40.0)	121.0 (38.4)	0.063
Apolipoprotein B (mg/dL)	96.0 (22.6)	84.4 (24.0)	0.004
Apolipoprotein A1 (mg/dL)	136.7 (18.5)	141.1 (19.7)	0.005
Insulin* (uUl/L)	9.6 (5.6, 11.9)	6.6 (4.2, 9.9)	0.003
HOMA-IR**	2.1 (1.3, 2.9)	1.5 (1.0, 2.3)	0.008
Adiponectin* (ng/dL)	11.8 (7.5, 18.9)	16.3 (11.5, 19.8)	0.012
Interleukin 6* (pg/dL)	2.5 (1.3, 4.0)	3.4 (2.0, 5.5)	0.007
C-reactive protein* (mg/dL)	0.370 (0.165, 0.617)	0.255 (0.070, 0.602)	0.036
Tumor necrosis factor α** (pg/dL)	10.9 (9.0, 13.4)	8.8 (7.2, 10.5)	0.003

*Log-transformed for analysis.

**Non-parametric test.

HOMA-IR: homeostatic model assessment for insulin resistance.

Genotype groups were in Hardy-Weinberg equilibrium, except the TNFα -308 G/A. The individuals with at least one variant allele were grouped and compared with those with the reference genotype.

### TNFα -308 G/A polymorphism

The overall frequency of the variant allele was 12.8% and the distribution of GG, GA, and AA genotypes was 79.4%, 15.4%, and 5.2%, respectively.

Variant A allele carriers did not improve values of waist circumference, HDL-c and Apo A1 after intervention but the reference genotype group did (Table 2). Regarding glucose metabolism, at baseline, higher fasting plasma glucose (103.9 ± 11.2 *versus* 97.8 ± 11.1 mg/dL; p = 0.012) and lower values of insulin [5.6 (4.0, 10.7) *versus* 9.8 (6.1, 12.0)] were observed in the A allele carriers and only this group decreased significantly fasting plasma glucose (−6.5 ± 11.8 *versus −*1.6 ± 14.1%; p = 0.050) after intervention. Logistic regression analysis showed that the presence of A allele was predictive of improvement in fasting plasma glucose after intervention, adjusted for sex and age (OR = 2.96, p = 0.025), but was not concerning other metabolic benefits. The significance of this result did not change when the presence of the other polymorphisms were entered in the model.

**Table 2:** Mean (SD) or median (interquartile interval) values of variables and variation after 9 months of lifestyle intervention according to SNP TNF −308 G/A genotypes

	**GG (n = 107)**			**AA + GA (n = 28)**		
	**Baseline**	**9 months**	**p**	**Baseline**	**9 months**	**p**
Energy intake (Kcal/day)	1851.6(685.0)	1555.4(580.6)	.001	1633.6(620.9)	1449.0(488.5)	.006
Total physical activity (min/wk)	167(60, 278)	205(75, 305)	.002	140(30, 210)	210(60, 400)	.021
Weight (kg)	79.0(15.4)	77.1(15.2)	.006	76.5(16.6)	74.8(16.9)	.006
Waist circumference (cm)	99.2(12.1)	98.3(11.7)	.029	98.0(13.0)	96.9(13.6)	.205
Fasting glucose (mg/dL)	97.8(11.1)	96.2(13.1)	.233	103.9(11.2)†	97.1(9.9)	.007
Triglycerides (mg/dL)	159.1(68.0)	154.2(74.6)	.428	138.2(56.2)	141.7(61.9)	.689
Total cholesterol (mg/dL)	200.9(42.8)	197.3(43.3)	.413	202.2(47.4)	197.1(45.9)	.559
HDL-c (mg/dL)	41.7(12.3)	46.7(13.2)	.020	45.5(12.7)	46.3(10.0)	.778
LDL-c (mg/dL)	127.3(38.9)	120.1(38.2)	.097	126.8(44.6)	122.4(41.3)	.647
Apolipoprotein B (mg/dL)	95.6(22.4)	84.8(23.8)	.004	95.3(23.4)	82.5(25.5)	.004
Apolipoprotein A1 (mg/dL)	135.8(17.8)	140.8(20.1)	.005	139.7(21.7)	142.0(19.0)	.443
Insulin* (uUl/L)	9.8(6.1, 12.0)	6.9(4.3, 10.5)	.009	5.6(4.0, 10.7)†	5.2(3.1, 8.8)	.151
Adiponectin* (ng/dL)	11.8(7.5, 18.7)	16.2(11.3, 19.4)	.019	11.7(7.3, 19.1)	16.2(12.2, 20.9)	.020
Interleukin 6* (pg/dL)	2.4(1.3, 3.7)	3.2(2.0, 5.2)	.013	2.4(1.1, 4.9)	4.2(2.4, 9.4)	.006
C-reactive protein* (mg/dL)	0.37(0.19, 0.59)	0.25(0.05, 0.55)	.001	0.36(0.13, 0.94)	0.32(0.15, 0.89)	.009
TNF-α** (pg/dL)	10.8(9.0, 13.2)	8.7(7.2, 10.8)	.020	11.3(9.4, 13.9)	8.9(6.9, 10.2)	.016

*log-transformed for analysis.

**Non-parametric test.

† p < 0.05 *versus* GG.

TNF-α: Tumor necrosis factor alpha.

Mean values of TNF-α and CRP decreased and IL-6 increased after intervention in both genotype groups.

### IL6 -174 G/C polymorphism

The overall frequency of the variant allele in sample was 46.2% and the distribution of GG, GC, and CC genotypes was 35.0%, 37.3%, and 27.6%, respectively.

Pre- and post-intervention mean body weight values were similar in genotype groups and waist circumference decreased significantly only in reference group (Table 3).

**Table 3:** Mean (SD) or median (interquartile interval) values of variables and variation after 9 months of intervention according to IL6 -174 G/C SNP genotypes

	**GG (n = 47)**			**GC + CC (n = 86)**		
	**Baseline**	**9 months**	**p**	**Baseline**	**9 months**	**p**
Energy intake (Kcal/day)	1748(729.6)	1481(588.0)	.002	1849(649.8)	1574(549.7)	.001
Total physical activity (min/wk)	140(60, 282)	240(70, 330)	.001	160(60, 260)	210(70, 290)	.001
Weight (kg)	76.9(15.3)	74.7(15.6)	.003	78.8(15.7)	77.2(15.3)	.001
Waist circumference (cm)	97.2(10.7)	95.7(11.1)	.036	99.4(12.8)	98.8(12.3)	.161
Fasting glucose (mg/dL)	99.4(11.4)	95.3(11.9)	.038	98.9(11.3)	97.1(12.8)	.233
Triglycerides (mg/dL)	161.1(60.3)	163.9(84.1)	.786	151.5(69.5)	145.3(65.1)	.299
Total cholesterol (mg/dL)	198.3(45.8)	209.5(42.7)	.122	202.9(42.9)	191.5(43.2)†	.011
HDL-c (mg/dL)	44.3(15.5)	45.6(13.6)	.446	41.6(10.5)	47.2(12.1)	.002
LDL-c (mg/dL)	123.1(42.7)	131.8(37.1)	.251	129.4(38.9)	115.5(38.6)†	.002
Apolipoprotein B (mg/dL)	95.6(23.2)	85.1(24.5)	.003	95.6(22.6)	84.1(24.0)	.001
Apolipoprotein A1 (mg/dL)	140.7(22.8)	143.6(24.7)	.197	134.3(15.9)	139.8(16.8)	.001
Insulin* (uUl/L)	8.1(5.4, 11.4)	5.7(4.0, 9.1)	.004	9.7(5.6, 12.1)	7.0(4.3, 10.6)	.002
Adiponectin* (ng/dL)	11.8(7.8, 19.3)	13.1(9.8, 19.7)	.091	12.1(7.3, 18.6)	16.8(12.4, 19.8)	.005
Interleukin 6* (pg/dL)	1.7(0.9, 3.5)	2.8(2.0, 5.4)	.007	2.5(1.4, 4.6)	3.6(2.0, 5.8)	.027
C-reactive protein* (mg/dL)	0.33(0.10, 0.59)	0.25(0.06, 0.64)	.005	0.37(0.19, 0.58)	0.25(0.07, 0.56)	.005
TNF-α** (pg/dL)	11.0(9.0, 13.7)	8.9(6.9, 10.5)	.017	11.0(9.4, 13.3)	8.8(7.2, 10.6)	.016

*Log-transformed for analysis.

**Non-parametric test.

† p < 0.05 *versus* GG.

TNF-α: Tumor necrosis factor alpha.

Variant C allele carriers showed greater improvement in the lipid profile after intervention than the reference genotype group. Lower concentrations of total cholesterol (191.5 ± 43.2 *versus* 209.5 ± 42.7 mg/dL, p = 0.023) and LDL-c (115.5 ± 38.6 *versus* 131.8 ± 37.1 mg/dL, p = 0.019) were observed only among the variants, who increased significantly Apo A1 concentrations (134.3 ± 15.9 to 139.8 ± 16.8 mg/dL, p = 0.001) and had a higher mean increment in HDL-c concentration (13.3 *versus* 3.0%, p = 0.043) than the reference group. On the other hand, changes in glucose metabolism were more favorable in the reference genotype. The latter group decreased fasting plasma glucose (99.4 ± 11.4 to 95.3 ± 11.9 mg/dL, p = 0.038), but not the variant allele carriers.

In both genotype groups, the intervention induced reductions in CRP and TNF-α concentrations and increase in mean IL-6 values, but only variant allele carriers showed significant increase in adiponectin concentration.

### Adipo 45 T/G polymorphism

The overall frequency of the variant allele was 14.7% and the TT, TG and GG genotypes were 72.0%, 26.5%, and 1.5%, respectively.

Variant G allele carriers showed a worse glucose and lipid metabolism response to intervention than the reference genotype. In contrast to reference genotype (Table 4), the variant allele group did not decrease mean values of fasting plasma glucose and Apo B nor increase HDL-c values.

**Table 4:** Mean (SD) or median (interquartile interval) values of variables and variation after 9 months of lifestyle intervention according to SNP AdipoQ 45 T/G genotypes

	**TT (n = 94)**			**TG + GG (n = 37)**		
	**Baseline**	**9 months**	**p**	**Baseline**	**9 months**	**p**
Energy intake (Kcal/day)	1805(720)	1558(617)	.001	1811(569)	1459(407)	.001
Total physical activity (min/wk)	150(60, 260)	210(60, 300)	.002	190(62, 280)	210(90, 360)	.008
Weight (kg)	79.4(17.1)	77.7(16.9)	.004	76.9(11.9)	74.9(12.1)	.003
Waist circumference (cm)	99.7(13.5)	98.9(13.1)	.077	97.9(8.8)	96.5(9.1)	.061
Fasting glucose (mg/dL)	99.2(11.8)	95.7(12.8)	.015	98.5(10.2)	98.8(11.3)	.891
Triglycerides (mg/dL)	155.5(65.9)	146.1(61.4)	.087	156.9(68.1)	166.7(95.3)	.432
Total cholesterol (mg/dL)	201.3(44.5)	195.3(43.6)	.167	197.7(42.6)	202.8(46.3)	.555
HDL-c (mg/dL)	42.2(12.4)	47.1(128.9)	.009	42.5(11.8)	44.6(10.8)	.242
LDL-c (mg/dL)	126.8(40.0)	118.9(38.1)	.068	125.1(41.8)	126.3(41.7)	.897
Apolipoprotein B (mg/dL)	96.6(22.5)	84.3(23.8)	.030	91.3(23.7)	82.9(26.0)	.068
Apolipoprotein A1 (mg/dL)	136.7(19.5)	140.6(20.9)	.013	135.9(17.0)	142.3(17.9)	.005
Insulin* (uUl/L)	10.1(6.0, 12.5)	7.0(4.3, 10.5)	.001	6.8(3.9, 10.5)	5.4(3.6, 9.2) †	.005
Adiponectin* (ng/dL)	11.2(7.5, 16.2)	15.0(11.1, 18.2)	.005	15.7(7.9, 22.4)	18.2(12.8, 22.4)	.060
Interleukin 6* (pg/dL)	2.4(1.3, 3.7)	3.4(2.2, 5.8)	.009	2.7(1.2, 4.6)	3.2(2.0, 5.1)	.398
C-reactive protein* (mg/dL)	0.33(0.13, 0.58)	0.29(0.07, 0.64)	.040	0.42(0.21, 0.80)	0.20(0.07, 0.48)	.001
TNF-α** (pg/dL)	10.8(8.9, 13.4)	9.1(7.4, 10.8)	.005	10.9(10.1, 13.2)	8.3(6.9, 10.0)	.004

*Log-transformed for analysis.

**Non-parametric test.

† p = 0.054 *versus* TT.

TNF-α: Tumor necrosis factor alpha.

In both genotype groups, the intervention induced reductions in CRP and TNF-α concentrations and increase in mean IL-6 values, but only variant allele carriers showed significant increase in adiponectin concentration significantly.

In order to evaluate the combined impact of the polymorphisms in glucose metabolism, the subset of 22 individuals with the three variants associated with worse glycemic response were grouped (TNFα -308 G + IL6 -174C + AdipoQ 45 G) and compared with the remainder. These groups had similar mean values of anthropometric and metabolic variables at baseline. After intervention, the group with worse glycemic response showed a significantly higher fasting plasma glucose levels (102.2 ± 11.6 *versus* 95.7 ± 11.4 mg/dL, p = 0.025), despite similar post-intervention anthropometric values.

## Discussion

Our data showed diverse responses to lifestyle intervention when analyzing the entire sample or subgroups stratified according to the SNPs. This may suggest a role for genotype in the response to lifestyle interventions. Contrasting with numerous reports on the frequencies of these three SNPs (TNF-α -308 G/A, IL6 -174 G/C and AdipoQ 45 T/G) associated with body adiposity, metabolic disturbances and inflammation, their association with response to intervention on lifestyle has been rarely investigated. Admixture of the Brazilian population does not allow adequate classification by ethnic group; this condition may limit the comparisons of our findings with the same polymorphisms studied in other populations. Despite the heterogeneity of our population, the frequencies of the variant alleles did not markedly differ from data previously reported in literature [39-41].

The TNF-α, IL-6 and AdipoQ polymorphisms were selected considering the participation of these cytokines in underlying mechanisms of obesity-related metabolic disturbances, such as inflammation and insulin resistance. It is consistently demonstrated the deleterious effect of TNF-α in insulin signaling pathways [11]. Both, TNF-α and IL-6 were shown to promote liver secretion of inflammatory biomarkers like CRP [42]. Also, insulin-sensitizing, anti-inflammatory and anti-atherogenic properties of adiponectin have been described [43]. Although it is clear that TNF-α and adiponectin exert opposite roles, findings regarding interleukins action are not so homogeneous.

The presence of the TNF-α -308 G/A polymorphism was associated with more favorable response of glucose metabolism to intervention, despite non-significant change in adiposity. Considering that the entire sample lose weight, it is possible that the subset with this TNF-α variant allele may have been protected against glucose disturbance independent of the body adiposity. At baseline, variant allele carriers had higher mean fasting plasma glucose levels, and exhibited a greater decrease after intervention compared with the reference group, even after adjustments. This is in line with a case control study in a Tunisian population, in which this SNP was not associated with obesity or T2D [16]. However, the results of a meta-analysis indicated no association with variant allele and fasting plasma glucose or T2D [44].

On the other hand, other intervention-induced cardiometabolic benefits, such as increases in HDL-c and Apo A1 concentrations, were not detected in variant allele carriers but in the reference genotype group. Deleterious effects of this TNF-α variant allele was previously suggested in a meta-analysis in which individuals carrying the -308 G/A polymorphism had greater risk of obesity and comorbidities [44].

It has been postulated that functional polymorphisms in promoter region could affect transcription rates and circulating levels of cytokines [45]. However, in our sample, the presence of the -308 G/A SNP did not influence TNF-α concentration at baseline or the response pattern to the intervention, since both genotype groups exhibited reductions in mean values of this cytokine. This is in agreement with a recent meta-analysis including healthy individuals that reported lack of associations between this TNF-α SNP and circulating levels, refuting a functional consequence of its presence [46].

In our study, some findings suggest that C allele carriers of the IL-6 -174 G/C polymorphism could have obtained more benefits from the intervention on lipid metabolism than those with the reference genotype, but not in glucose metabolism. Only C allele carriers showed a significant decrease in total cholesterol and LDL-c and increase in HDL-c and Apo A1 concentrations. Cross-sectional studies, conducted in several populations, had already reported a worse lipid profile in the reference genotype individuals compared with variant carriers. In Caucasian populations, significantly higher plasma triglyceride, total cholesterol and LDL-c and slightly lower HDL-c were observed in individuals with the reference genotype than in C variant allele carriers [17,47]. However, the variant allele carriers did not reduce significantly fasting plasma glucose but did those with reference genotype. These contrasting results might be at least in part to a decrease in central adiposity verified only in the reference genotype group. Our findings are in agreement with those obtained from a Finnish Caucasian population in which GG genotype had lower fasting plasma glucose than C allele carriers [17].

Both genotype groups IL-6 -174 G/C SNP responded to the intervention with reductions in plasma TNF-α and CRP, but with unexpected elevations in mean IL-6 concentrations. Additionally, variant allele carriers had significant increase adiponectin concentration after intervention. Attenuation of pro-inflammatory actions of TNF-α and CRP and the favorable effect of adiponectin on inflammation and insulin sensitivity, those findings are desirable for individuals at high cardiometabolic risk undergoing lifestyle interventions.

To our knowledge, increased circulating levels of IL-6, particularly in carriers of IL-6 -174 G/C SNP submitted to long-term lifestyle intervention, have not been reported. It is known that IL-6 is released from skeletal muscle during exercise proportionally to exercise intensity [48]. However, the increased output found in the recovery phase of prolonged exercise is most probably from the skeletal muscle and, to a lesser extent, from the subcutaneous adipose tissue [49]. Our intervention program included physical activity but the majority of the sample performed light-to-moderate exercises (data not shown). Given the fact that IL-6, more than any other cytokine, is produced locally in the skeletal muscle in response to exercise, it is likely that it plays a beneficial role in mediating exercise-related metabolic changes [50].

Our recommendation to enhance physical activity during the intervention was attended by the entire sample since the proportion of active individuals increased at the end of the intervention. Despite no correlation detected between changes in physical activity and changes in IL-6 concentration (data not shown), we speculate that the IL-6 elevation in our sample could be partially attributed to exercise. This finding does not seem to reflect a pro-inflammatory effect, since mean values of CRP reduced significantly. Further investigations, including a large spectrum of interleukins, are necessary to understand their role in interventions on life habits.

The group of carriers of variant allele AdipoQ 45 T/G SNP did not change fasting plasma glucose and adiponectin concentrations but the reference genotype group did. We speculate that the lack of increment in adiponectin concentration after intervention may have contributed to impede benefits in glucose metabolism in the variant allele carriers. Controversial data have been reported among populations [27,29,51]. Our result may be considered somehow different from a case–control study, since higher adiponectin concentration was found among variant allele homozygous in healthy Korean woman [51]. In contrast, a cross-sectional survey in Chinese population showed lower plasma adiponectin in variant allele carriers compared with the TT genotype [27]. Data from the STOP-NIDDM trial [29] and a case control study in obese Iranian population [52] are in line with ours, since they support that variant G allele might confer susceptibility to T2D.

Our findings indicated unfavorable effects of the variant allele of the AdipoQ 45 T/G in lipid profile, when compared to the entire sample and to the reference genotype group. Carriers of the G allele did not decrease Apo B nor increase HDL-c concentrations after intervention. Similarly to ours, in a Chinese cross-sectional study, G carriers had higher total and LDL-c levels than the reference genotype group [27]. The beneficial response of CRP concentrations to our intervention seems having not depended on the AdipoQ 45 T/G SNP. This set of evidence suggests the presence of this SNP impedes metabolic response to lifestyle interventions.

The comparison of the group the individuals with the three variants associated with worse glycemic response with the remainder sample could suggest that the combination of polymorphisms would enhance the negative impact on glucose metabolism. Despite similar fasting plasma glucose at baseline, the former group finished the intervention with higher levels, which would be compatible with this hypothesis. This speculation deserves further investigation based on large samples.

Despite the strength of addressing important SNPs codifying for proteins known to affect inflammatory status and insulin sensitivity, and examining a great number of metabolic biomarkers, our study has the limitation of the sample size. The small sample size could be responsible for the absence of Hardy-Weinberg equilibrium for TNF -308 G/A. Actually, a study including a large sample of individuals from the same region of Brazil indicated that this SNP is in HWE [53]. Another possible explanation for such finding could be that this SNP itself would be a risk factor for diabetes and cardiometabolic risk. Therefore, our results should be considered preliminary at best.

In summary, the TNFα -308 G/A SNP may predispose a better response of glucose metabolism to intervention. The IL-6 -174 G/C SNP may confer a beneficial effect on lipid but not in glucose metabolism. Our findings reinforce unfavorable effects of the AdipoQ 45 T/G SNP in lipid profile and glucose metabolism after lifestyle intervention in Brazilians at cardiometabolic risk. Further studies on SNPs of genes involved on the control of inflammatory status and insulin sensitivity should provide more subsidies to achieve the goals of preventing metabolic disturbances through lifestyle changes in individuals at high cardiometabolic risk.

## Competing interests

The authors declare no competing interests.

## Authors’ contributions

MLRC, CRB, MMP and ASC have collected the research data. MLRC wrote, reviewed and edited the data. MMR reviewed the manuscript. SRGF reviewed the manuscript and was a coordinator of the research group. All authors read and approved the final manuscript.
